# Correction: Cellular repressor of E1A-stimulated genes 1 enhances skeletal muscle performance through the stimulation of muscle differentiation and Akt-mTOR signaling pathway activation

**DOI:** 10.1371/journal.pone.0356625

**Published:** 2026-08-18

**Authors:** Ayumi Goto, Michihiro Hashimoto, Sho Yokogawa, Yuzu Naruse, Hitoshi Yamashita

Fig 2D is a duplicate of Fig 2E. The authors have provided a corrected version of Fig 2 here.

**Fig 2:**
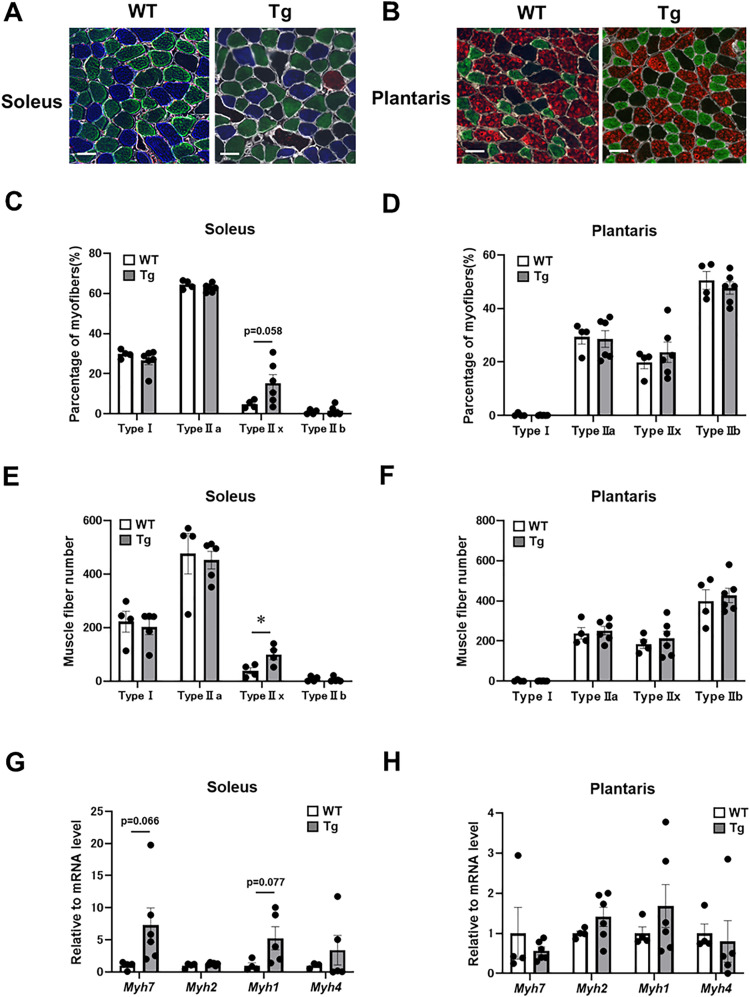
Muscle fiber-type composition and myosin heavy chain isoforms in wild-type (WT) and adipocyte P2-CREG1-transgenic (Tg) mice. Muscle fiber-type composition was analyzed in the soleus and plantaris muscles of 5-month-old WT and Tg mice. (A-B) Immunostaining of myofibers in the soleus (A) and plantaris (B) muscles of WT and Tg mice. Type I (blue), type IIa (green), type IIx (black), type IIb (red), and lamin (white). Scale bar, 50 µm. (C-D) Distribution of muscle fiber types in the soleus (C) and plantaris (D) muscles. (E-F) Number of myofibers in the soleus (E) and plantaris (F) muscles of WT and Tg mice. (G-H) Relative levels of Myh7, Myh2, Myh1 and Myh4 mRNA in the soleus (G) and plantaris (H) muscles. Data were normalized to the 36b4 mRNA level. Data are presented as mean ± SEM; n = 4–6 per group. A student’s t-test was performed; *P < 0.05, versus WT.
