# Supplementary material for: Cellular repressor of E1A-stimulated genes 1 enhances skeletal muscle performance through the stimulation of muscle differentiation and Akt-mTOR signaling pathway activation
Source: PLoS One. 2025 Jul 17;20(7):e0328485. doi: 10.1371/journal.pone.0328485 (PMC12270121; doi:10.1371/journal.pone.0328485)
Supplement: S1 Raw images — (PDF) [file pone.0328485.s001.pdf]

Original images for Immunohistochemistry

Cellular repressor of E1A-stimulated genes 1 enhances skeletal muscle performance through the stimulation of muscle differentiation and Akt-mTOR signaling pathway activation.

Ayumi Goto, Michihiro Hashimoto, Sho Yokogawa,
Yuzu Naruse, Hitoshi Yamashita

Soleus

WT

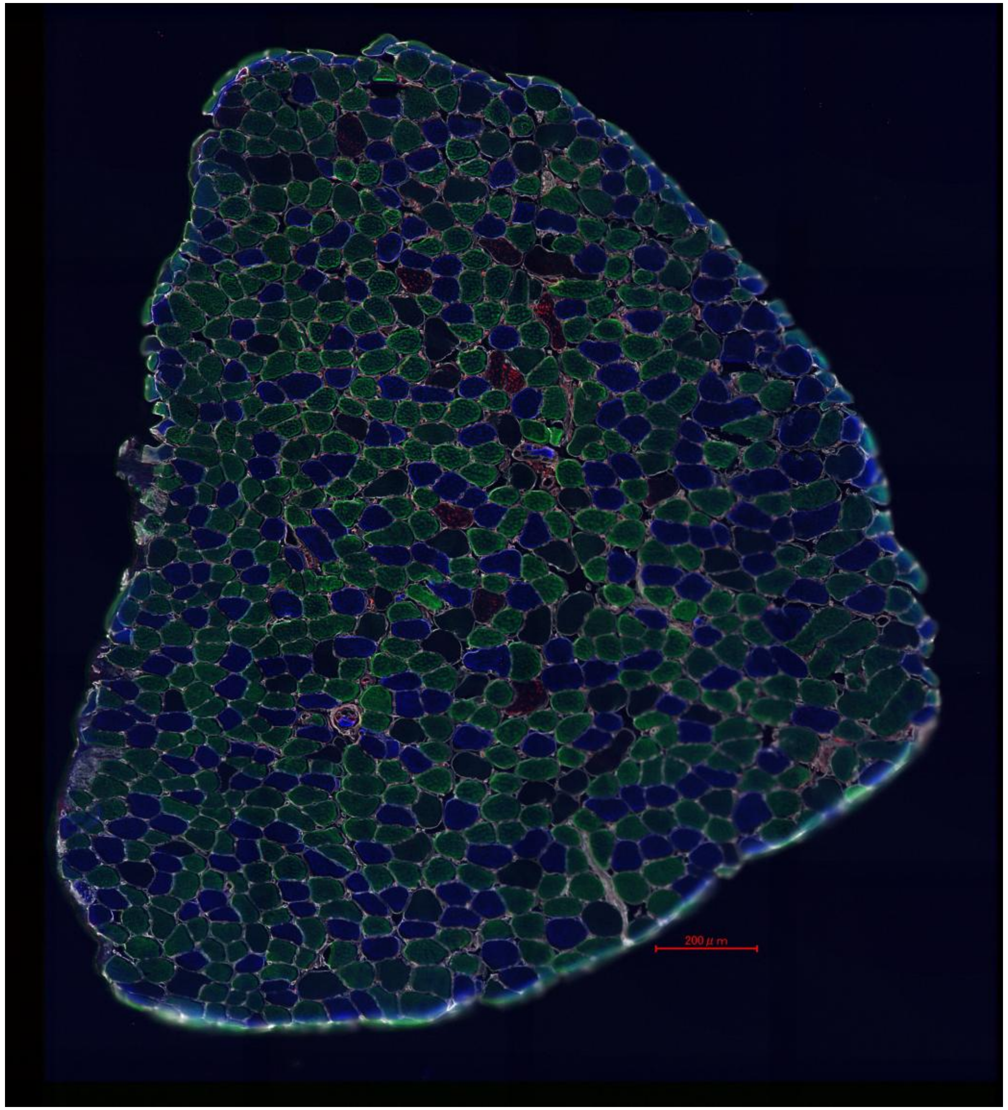

Tg

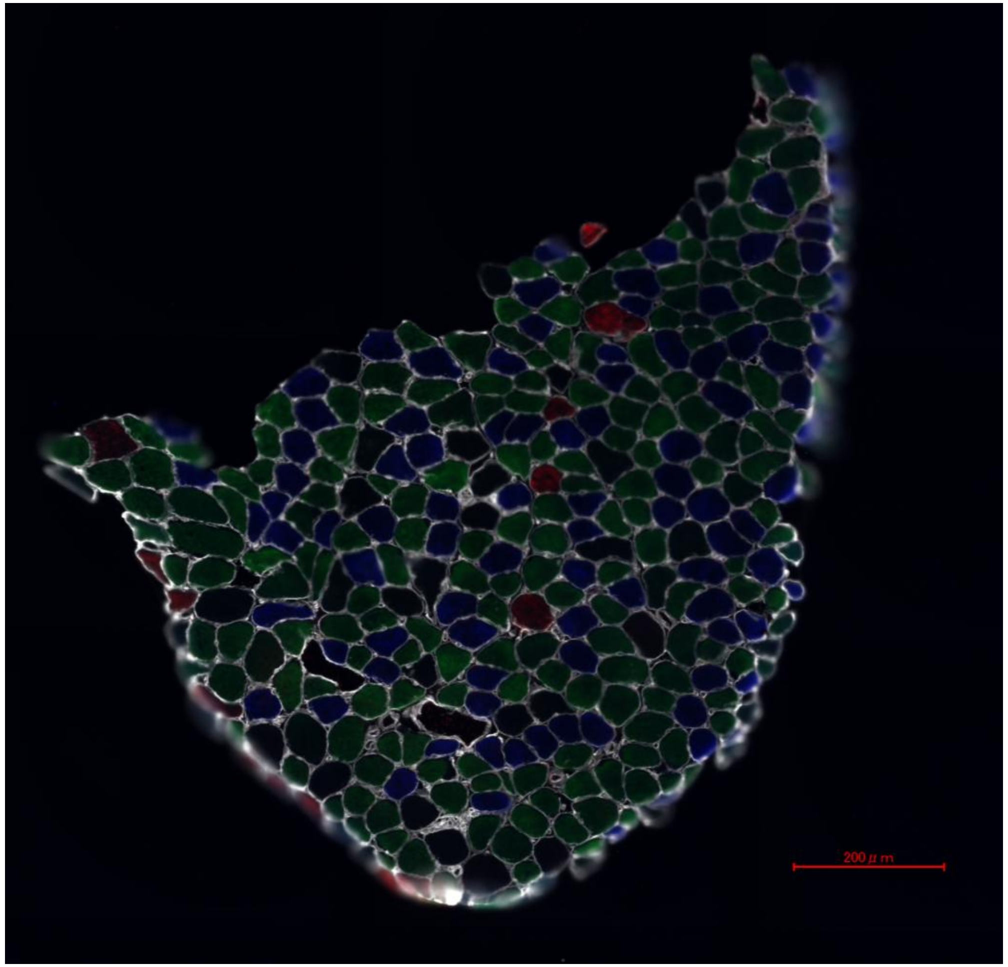

Supporting information

Plantaris

WT

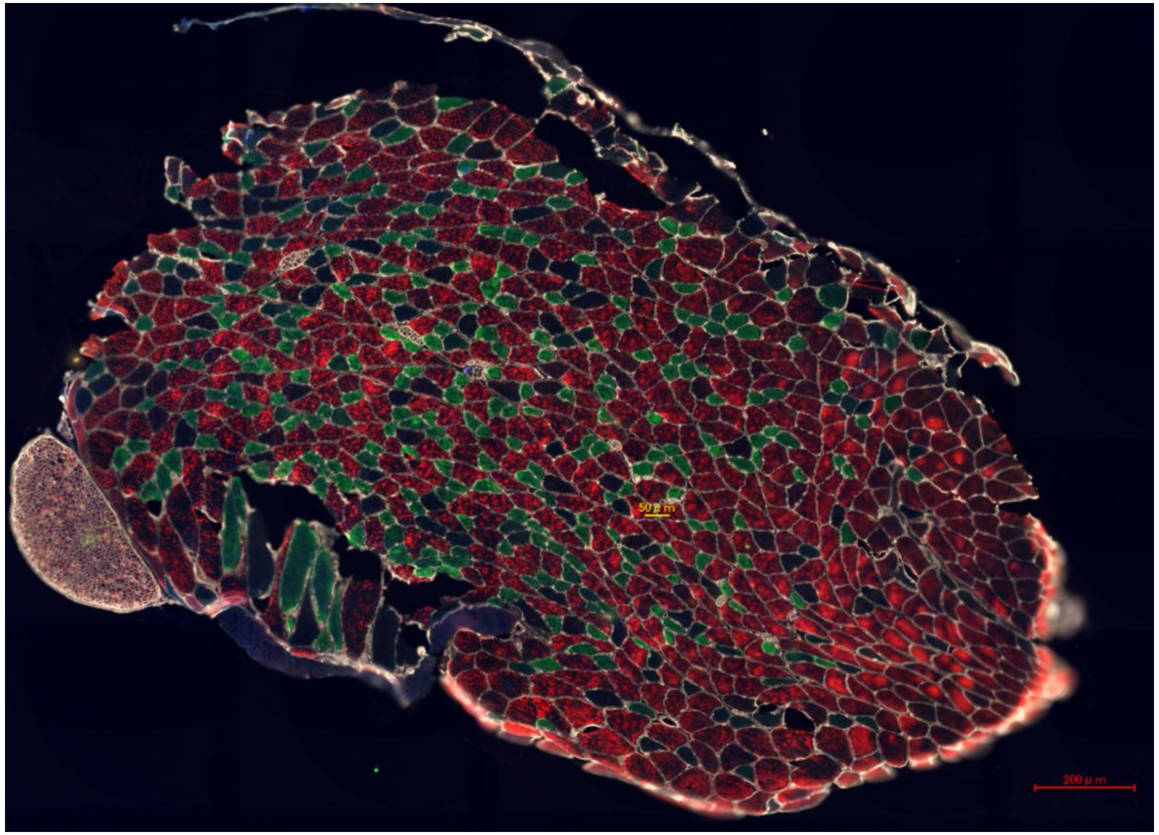

Tg

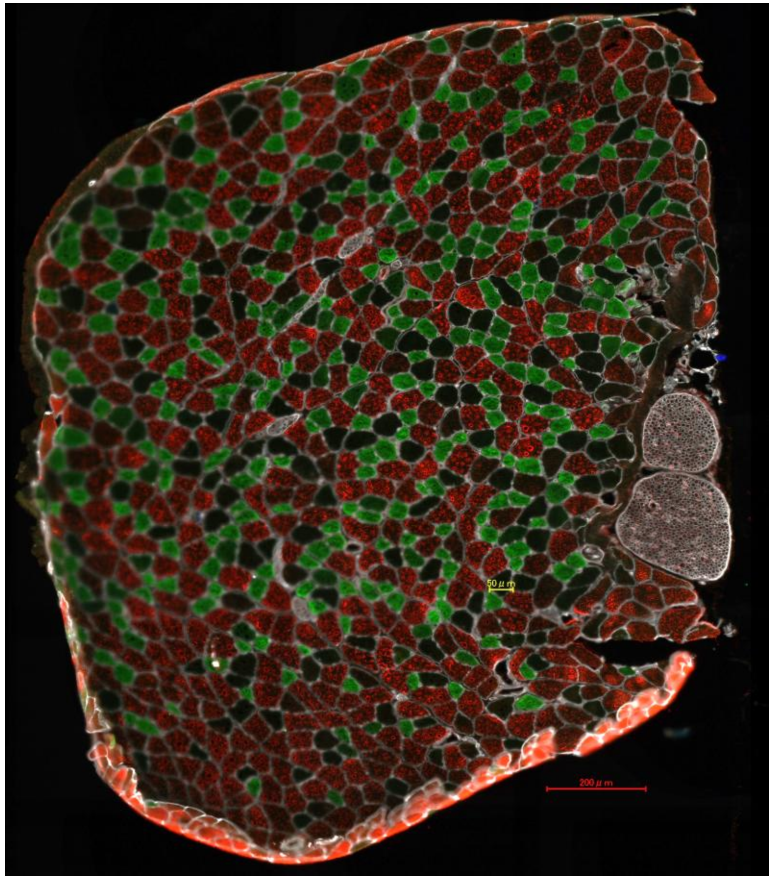

Supporting information

Original images for western blotting

Cellular repressor of E1A-stimulated genes 1 enhances skeletal muscle performance through the stimulation of muscle differentiation and Akt-mTOR signaling pathway activation.

Ayumi Goto, Michihiro Hashimoto, Sho Yokogawa,
Yuzu Naruse, Hitoshi Yamashita

Serum CREG1

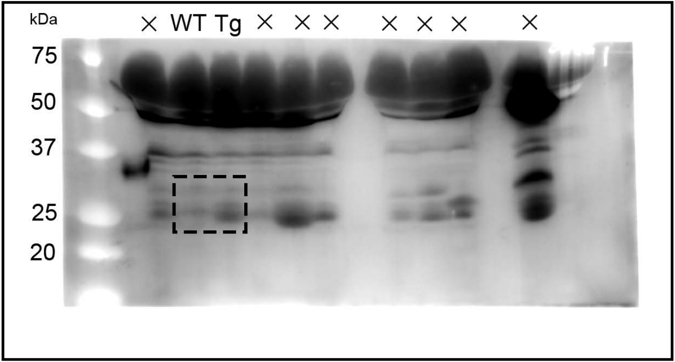

Supporting information

CREG1

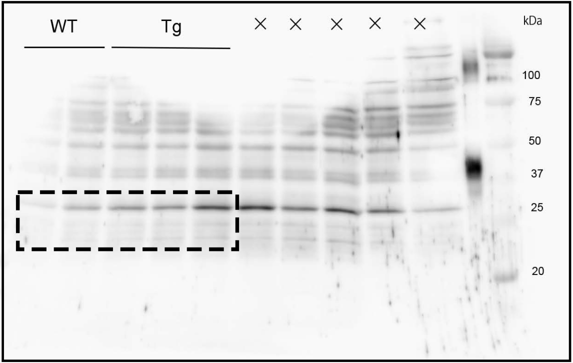

Supporting information

GAPDH

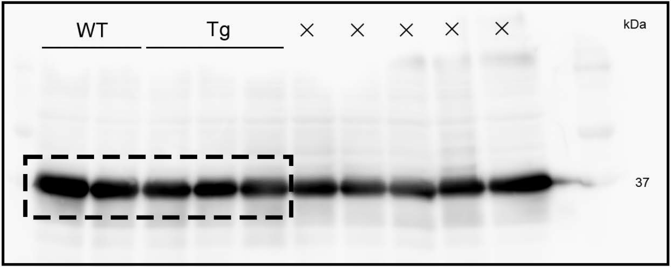

Supporting information

IGF2R

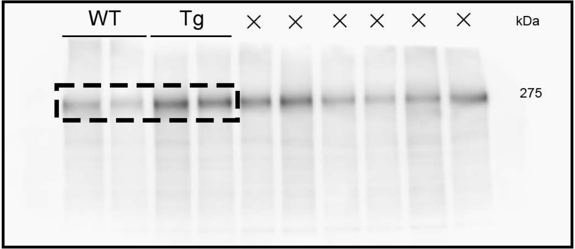

Supporting information

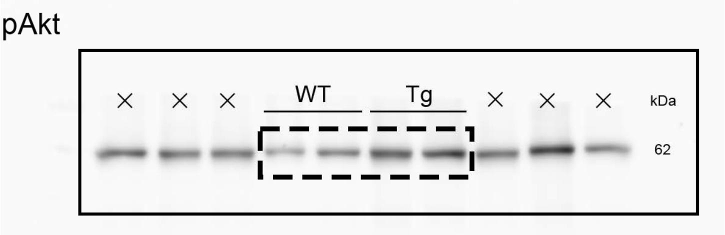

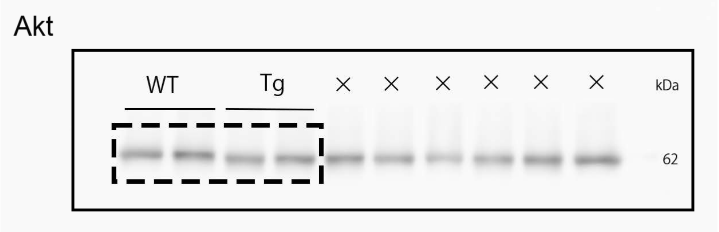

Supporting information

pmTOR

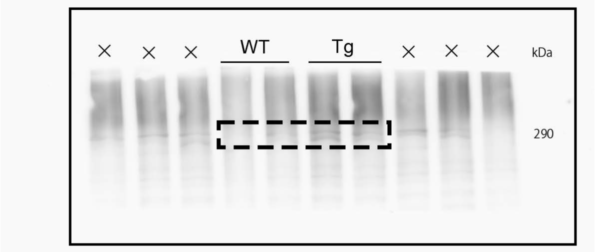

mTOR

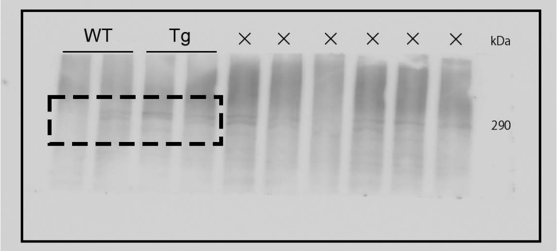

Supporting information

GAPDH

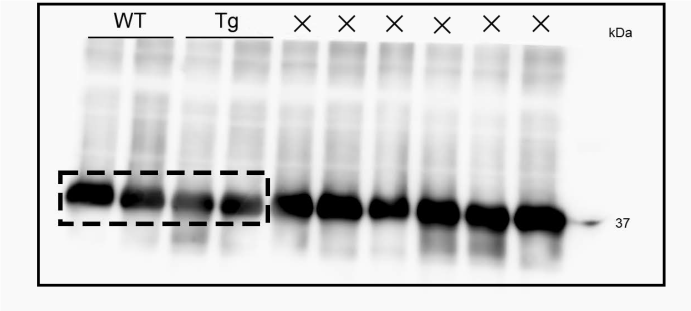

Supporting information

CREG1

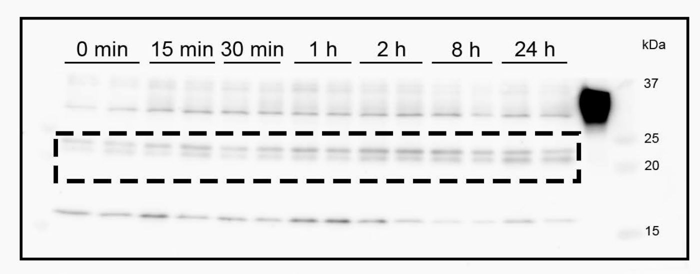

Tubulin

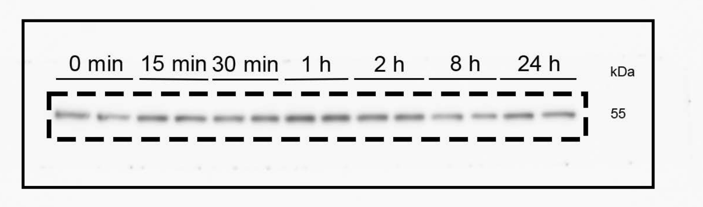

Supporting information

pAkt

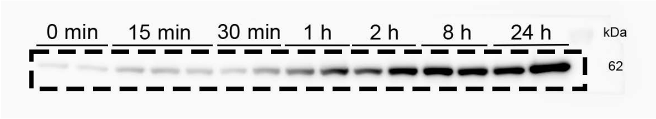

Akt

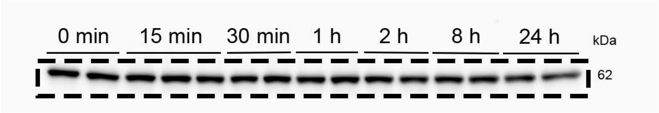

Supporting information

pmTOR

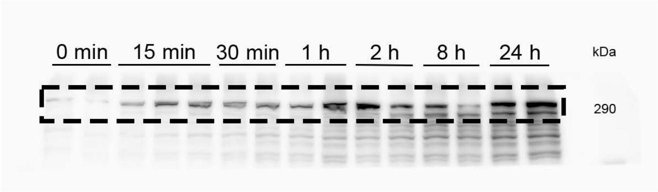

mTOR

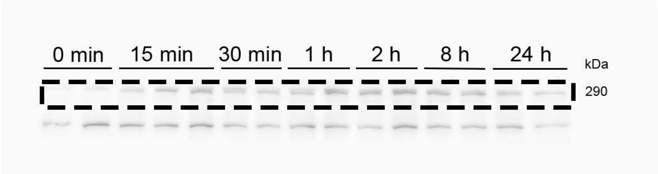

Supporting information
