# Supplementary material for: Cellular repressor of E1A-stimulated genes 1 enhances skeletal muscle performance through the stimulation of muscle differentiation and Akt-mTOR signaling pathway activation
Source: PLoS One. 2025 Jul 17;20(7):e0328485. doi: 10.1371/journal.pone.0328485 (PMC12270121; doi:10.1371/journal.pone.0328485)
Supplement: File S1 — Table S2. Data analysis results for Figure 1 using Student’s t-test. Table S3. Data for Figure 2C. Table S4. Data analysis results for Figure 2C using Student’s t-test. Table S5. Data for Figure 2D. Table S6. Data analysis results for Figure 2D using Student’s t-test. Table S7. Data for Figure 2E. Table S8. Data analysis results for Figure 2E using Student’s t-test. Table S9. Data for Figure 2F. Table S10. Data analysis results for Figure 2F using Student’s t-test. Table S11. Data for Figure 2G. Table S12. Data analysis results for Figure 2G using Student’s t-test. Table S13. Data for Figure 2H. Table S14. Data analysis results for Figure 2H using Student’s t-test. S15. Data for Figure 3A-C. Table S16. Data analysis results for Figure 3A-C using Student’s t-test. Table S17. Data for Figure 3D. Table S18. Data analysis results for Figure 3D using Student’s t-test. Table S19. Data for Figure 4A-B. Table S20. Data analysis results for Figure 4A-B using one-way ANOVA with post-hoc Tukey–Kramer test. Table S21. Data for Figure 4C-D. Table S22. Data analysis results for Figure 4C-D using one-way ANOVA with post-hoc Tukey–Kramer test. Table S23. Data for Figure 5A-C. Table S24. Data analysis results for Figure 5A-C using one-way ANOVA with post-hoc Tukey–Kramer test. (PDF) [file pone.0328485.s002.pdf]

Table S1. Data for Figure 1.

	**WT**	**Tg**
Mean	0.034	0.039
SEM	0.001	0.001

Table S2. Data analysis results for Figure 1 using Student's *t*-test.

	***p*-value**
WT vs. Tg	0.0099

Table S3. Data for Figure 2C.

	Type Ⅰ		Type Ⅱa		Type Ⅱx		Type Ⅱb	
	WT	Tg	WT	Tg	WT	Tg	WT	Tg
Mean	29.80	26.63	64.37	62.72	4.88	15.30	0.95	1.47
SEM	0.92	2.17	1.05	0.82	0.99	4.26	0.56	0.93

Table S4. Data analysis results for Figure 2C using Student's *t*-test.

	Type Ⅰ	Type Ⅱa	Type Ⅱx	Type Ⅱb
WT vs. Tg	0.266	0.492	0.058	0.818

Table S5. Data for Figure 2D.

	Type Ⅰ		Type Ⅱa		Type Ⅱx		Type Ⅱb	
	WT	Tg	WT	Tg	WT	Tg	WT	Tg
Mean	0.30	0.09	29.39	28.64	19.86	23.65	50.46	47.62
SEM	0.30	0.05	2.66	3.11	2.37	3.82	3.34	2.29

Table S6. Data analysis results for Figure 2D using Student's *t*-test.

	Type Ⅰ	Type Ⅱa	Type Ⅱx	Type Ⅱb
WT vs. Tg	0.545	0.870	0.481	0.487

Table S7. Data for Figure 2E.

	Type Ⅰ		Type Ⅱa		Type Ⅱx		Type Ⅱb	
	WT	Tg	WT	Tg	WT	Tg	WT	Tg
Mean	221.8	201.1	476	452	38.3	98.8	8.0	5.2
SEM	38.99	28.54	75.96	32.94	11.53	18.86	4.78	4.02

Table S8. Data analysis results for Figure 2E using Student's *t*-test.

	Type Ⅰ	Type Ⅱa	Type Ⅱx	Type Ⅱb
WT vs. Tg	0.682	0.763	0.034	0.665

Table S9. Data for Figure 2F.

	Type Ⅰ		Type Ⅱa		Type Ⅱx		Type Ⅱb	
	WT	Tg	WT	Tg	WT	Tg	WT	Tg
Mean	2.3	0.8	238	252	185	213	399	427
SEM	2.25	0.40	28.93	21.24	21.84	35.82	56.97	35.23

Table S10. Data analysis results for Figure 2F using Student's *t*-test.

	Type Ⅰ	Type Ⅱa	Type Ⅱx	Type Ⅱb
WT vs. Tg	0.465	0.710	0.580	0.667

Table S11. Data for Figure 2G.

	Myh7		*Myh2*		*Myh1*		*Myh4*	
	WT	Tg	WT	Tg	WT	Tg	WT	Tg
Mean	1.00	7.30	1.00	1.26	1.00	5.26	1.00	3.41
SEM	0.31	2.69	0.19	0.08	0.42	1.62	0.21	2.10

Table S12. Data analysis results for Figure 2G using Student's *t*-test.

	Myh7	*Myh2*	*Myh1*	*Myh4*
WT vs. Tg	0.066	0.185	0.077	0.463

Table S13. Data for Figure 2H.

	Myh7		*Myh2*		*Myh1*		*Myh4*	
	WT	Tg	WT	Tg	WT	Tg	WT	Tg
Mean	1.00	0.55	1.00	1.41	1.00	1.68	1.00	0.67
SEM	0.65	0.10	0.06	0.24	0.16	0.53	0.23	0.47

Table S14. Data analysis results for Figure 2H using Student's *t*-test.

	Myh7	*Myh2*	*Myh1*	*Myh4*
WT vs. Tg	0.541	0.148	0.268	0.756

Table S15. Data for Figure 3A-C.

	Serum CREG1		*CREG1 mRNA*		CREG1	
	WT	Tg	WT	Tg	WT	Tg
Mean	1.00	1.44	1.00	4.06	1.00	2.54
SEM	0.25	0.20	0.14	0.32	0.09	0.21

Table S16. Data analysis results for Figure 3A-C using Student's *t*-test.

	Serum CREG1	*CREG1 mRNA*	CREG1
WT vs. Tg	0.201	< 0.001	0.024

Table S17. Data for Figure 3D.

	IGF2R		pAkt		Akt		pmTOR		mTOR	
	WT	Tg	WT	Tg	WT	Tg	WT	Tg	WT	Tg
Mean	1.00	2.10	1.00	1.87	1.00	1.22	1.00	1.80	1.00	1.99
SEM	0.15	0.42	0.08	0.22	0.22	0.10	0.32	0.13	0.19	0.22

Table S18. Data analysis results for Figure 3D using Student's *t*-test.

	IGF2R	pAkt	Akt	pmTOR	mTOR
WT vs. Tg	0.048	0.031	0.307	0.020	0.029

Table S19. Data for Figure 4A-B.

		**0 d**	**1 d**	**2 d**	**3 d**	**4 d**	**5 d**
Creg1	Mean	1.00	2.65	2.81	2.81	1.74	2.37
	SEM	0.04	0.15	0.06	0.08	0.02	0.09
Myod1	Mean	1.00	1.19	2.23	3.99	3.59	2.01
	SEM	0.11	0.09	0.32	0.34	1.01	0.09

Table S20. Data analysis results for Figure 4A-B using one-way ANOVA with post-hoc Tukey–Kramer test.

	Creg1	***Myod1***
0 d vs. 1 d	< 0.001	1.000
0 d vs. 2 d	< 0.001	0.449
0 d vs. 3 d	< 0.001	< 0.001
0 d vs. 4 d	< 0.001	0.002
0 d vs. 5 d	< 0.001	0.637

Table S21. Data for Figure 4C-D.

		**Con siRNA**	**Creg1 siRNA**	**Creg1 siRNA**
	CREG1-MH	-	+	+
Creg1	Mean	1.00	0.37	0.39
	SEM	0.09	0.06	0.04
Myod1	Mean	1.00	0.82	1.05
	SEM	0.04	0.06	0.05

Table S22. Data analysis results for Figure 4C-D using one-way ANOVA with post-hoc Tukey–Kramer test.

	Creg1	***Myod1***
Con siRNA(-) vs. Creg1 siRNA (-)	0.0013	0.057
Con siRNA(-) vs. Creg1 siRNA (+)	0.0016	0.730
Creg1 siRNA(-) vs. Creg1 siRNA (+)	0.974	0.023

Table S23. Data for Figure 5A-C.

		0 min	15 min	30 min	1 h	2 h	8 h	24 h
**CREG1**	Mean	1.00	1.19	0.99	1.27	1.91	2.13	2.10
	SEM	0.21	0.28	0.11	0.09	0.01	0.43	0.08
**pAkt**	Mean	1.00	3.56	4.55	14.19	22.73	30.53	40.56
	SEM	0.13	0.21	1.81	2.82	6.55	0.09	8.40
**pmTOR**	Mean	1.00	1.82	1.79	1.97	2.66	2.18	7.06
	SEM	0.22	0.22	0.07	0.59	0.68	0.67	2.07

Table S24. Data analysis results for Figure 5A-C using one-way ANOVA with post-hoc Tukey–Kramer test.

	CREG1	pAkt	pmTOR
0 min vs. 15 min	0.641	0.998	0.984
0 min vs. 30 min	0.862	0.993	0.992
0 min vs. 1 h	0.527	0.328	0.977
0 min vs. 2 h	0.042	0.047	0.795
0 min vs. 8 h	0.017	0.009	0.945
0 min vs. 24 h	0.019	0.001	0.011
