# Supplementary material for: Cellular repressor of E1A-stimulated genes 1 enhances skeletal muscle performance through the stimulation of muscle differentiation and Akt-mTOR signaling pathway activation
Source: PLoS One. 2025 Jul 17;20(7):e0328485. doi: 10.1371/journal.pone.0328485 (PMC12270121; doi:10.1371/journal.pone.0328485)
Supplement: Attachment — Submitted filename: Response to reviewers 20250625.docx [file pone.0328485.s005.docx]

Reviewer #2: This reviewer appreciates the revisions to the manuscript. However, one issue remains -- analysis of the wire hanging test. If statistics cannot be performed on the data in Fig 1B, then it is not appropriate to conclude that the WT is different than Tg group. By convention in rigorous scientific literature, quantitative data is not considered different unless it is statistically different. Therefore, this data should additionally be presented in a manner where proper statistics can be calculated, for example as was done in Ref #23, Egawa et al. Then, conclusions throughout the manuscript regarding the wire hang test must be based only on data where statistics are performed.

We thank the reviewer for these insightful comments. We used the analysis methods from previous study (Raymackers JM, et al. (2003)) to present Figure 1b. Following your suggestion, we reanalyzed the data using Egawa et al.’s method and found no significant differences in the longest holding time or holding impulse. Therefore, we have removed Figure 1b and the description of wire hanging in the text. Changes have been marked in red in the “Revised Article with Changes Highlighted” file.
